# Adjuvant Radiation Therapy of Retroperitoneal Sarcoma: The Role of Intraoperative Radiotherapy (IORT)

**DOI:** 10.1155/S1357714X00000037

**Published:** 2000-03

**Authors:** Kenneth S. hu, Louis C. Harrison

**Affiliations:** 1 The Charles and Bernice Blitman Department of Radiation Oncology Beth Israel Medical Center NewYork USA; 2 St Lukes-Roosevelt Hospital Center NewYork USA

## Abstract

*Purpose.* The purpose is to review the natural history, the clinicopathological prognostic factors, and the role of adjuvant radiation therapy with particular attention to the limited but favorable experience with IORT.

*Methods.* Retroperitoneal sarcomas present a continuing therapeutic challenge to the oncologist. In contrast to sarcomas of the extremity and superficial trunk in which complete resection plus radiation therapy results in excellent local control, sarcomas of the retroperitoneum are difficult to resect and even if completely resected, demonstrate high rates of local relapse, the primary pattern of failure. Due to the proximity of normal organs, the delivery of therapeutic doses of adjuvant external beam radiation therapy is problematic.To deliver adequate doses (>60 Gy) of external beam to most patients would result in unacceptable toxicity. The therapeutic dilemma is unfortunate and better strategies are needed. One attractive approach has been to incorporate intraoperative radiation therapy (IORT) with maximal resection and external beam radiation.

* Results and Discussion. * A number of institutions have explored this approach with encouraging preliminary results.


Sarcoma (2000) **4**, 11–16

ORIGINAL ARTICLE

### Adjuvant radiation therapy of retroperitoneal sarcoma: the role of intraoperative radiotherapy (IORT)

KENNETH S. HU[1] & LOUIS B. HARRISON[1,2]

*[1]The Charles and Bernice Blitman Department of Radiation Oncology, Beth Israel Medical Center, and [2]St Lukes-Roosevelt Hospital Center, New York, USA*

**Abstract**

*Purpose.* The purpose is to review the natural history, the clinicopathological prognostic factors, and the role of adjuvant radiation therapy with particular attention to the limited but favorable experience with IORT.

*Methods.* Retroperitoneal sarcomas present a continuing therapeutic challenge to the oncologist. In contrast to sarcomas of the extremity and superficial trunk in which complete resection plus radiation therapy results in excellent local control, sarcomas of the retroperitoneum are difficult to resect and even if completely resected, demonstrate high rates of local relapse, the primary pattern of failure. Due to the proximity of normal organs, the delivery of therapeutic doses of adjuvant external beam radiation therapy is problematic. To deliver adequate doses (>60 Gy) of external beam to most patients would result in unacceptable toxicity. The therapeutic dilemma is unfortunate and better strategies are needed. One attractive approach has been to incorporate intraoperative radiation therapy (IORT) with maximal resection and external beam radiation.

*Results and Discussion.* A number of institutions have explored this approach with encouraging preliminary results.

**Key words:** *retroperitoneal sarcoma, intraoperative radiation therapy (IORT), high-dose rate IORT, electron beam, complications*

### Introduction

Retroperitoneal sarcomas present a continuing challenge to the oncologist. With only 1000 cases diagnosed per year in the USA, they are a rare tumor, comprising about 15% of all sarcomas and 40% of all primary retroperitoneal tumors.[1] Their rarity impedes data collection and limits the power of adequately designed trials. In contrast, the published data with extremity sarcoma is more extensive. It has been clearly documented that complete resection plus radiation therapy can locally control the overwhelming majority of extremity sarcomas and superficial trunk.[2–19] In this regard, the radiation therapy approach can be either by external beam, brachytherapy or a combination of both, yielding local control rates of 80–90% and 5-year overall survival of 80%.[7,8,16,20] Sarcomas of the retroperitoneum have been a far different story. Due to its remote location with multiple adjacent critical organs, sarcomas arising from this location present as large, advanced stage tumors that are difficult to resect with adequate margin. The proximity of normal organs such as viscera and neurovascular structures has made the delivery of therapeutic doses of postoperative external beam irradiation problematic. To deliver adequate doses (>60 Gy) of external beam radiation would result in unacceptable toxicity. The therapeutic dilemma is unfortunate and better strategies are needed. One attractive approach has been to incorporate intraoperative radiation therapy using either electrons (IOERT) or high-dose rate photons delivered via remote afterloading (HDR-IORT) with maximal resection and external beam radiation. The purpose of this review is to overview the need for effective adjuvant radiation after maximal resection of retroperitoneal sarcomas and to report the limited but encouraging experience using IORT in combination with conventional external beam radiation.

### Clinicopathological prognostic factors

Achieving a gross total resection (GTR) of either a primary or recurrent retroperitoneal sarcoma is by far the most important predictive factor for local control and survival.[1,21–24] When GTR is achieved, 5-year overall survival is about 50% (range 35–74%, Table 1) Moreover, after GTR, the microscopic residual disease status appears important as patient with microscopic disease present appear to have worse survival[25] and/or local control[22] compared to those with negative residual. The outlook for patients with

Correspondence to: Kenneth S. Hu, MD, Attending, The Charles and Bernice Blitman, Department of Radiation Oncology, Beth Israel Medical Center, 10 Union Square East, New York, NY 10003, USA. Tel: 212 844-8022; Fax: 212 844-8086; khu@bethisraelny.org

1357-714Xprint/1369-1643online/00/010011-06 © 2000 Taylor & Francis Ltd

**Table 1**. *Local control and survival after gross total resection of retroperitoneal sarcomas*

| | Tx Date | No. of patients | Med F/U (years) | %HG | %RT (median dose) | 5-year LR (actuarials %) | 5-year OS (actuarials) % |
|---|---|---|---|---|---|---|---|
| MSKCC[30] | 1982–97 | 185* | 2.3 | 58 | NS | 41 | 70 |
| MSKCC[21] | 1982–87 | 67 | 2.6 | 55 | NS | 49 | 74 |
| U Fla[27] | 1970–94 | 49 | NS | 54 | NS | 46 (crude) | 58 |
| MSKCC[32] | 1951–77 | 47 | NS | NS | 40 (NS) | 77 | 40 |
| PMH[23] | 1975–88 | 45 | 6.3 | NS | 80 (40 Gy) | 50 (10 year 82%) | 55 (10 year 22%) |
| Univ. Minn.[37] | 1941–87 | 31 | 11 | 77 | NS | NS | 48 |
| Netherlands[24] | 1973–90 | 29 | 3.2 | 44 | 45 | 63 | 35 |
| Roswell Park[6] | 1957–80 | 27 | NS | NS | NS | NS | 64 |
| MCV[26] | 1964–82 | 18 | NS | NS | 33 (NS) | 56 (crude) | 70 |

HG=high grade, LR=local recurrence, OS=overall survival, NS=not stated, MSKCC=Memorial Sloan-Kettering Cancer Center, PMH=Princess Margaret Hospital, MCV=Medical College of Virginia, *All patients presented with primary tumors.

gross residual disease is dismal with 5-year survival of less than 5%.[21,26] Given the importance of achieving GTR, aggressive surgery must be pursued. To obtain a maximal en bloc resection, it is common to resect abutting organs.[21,27] In the Memorial Sloan-Kettering Cancer Center (MSKCC) series, Jacques demonstrated that that resection of adjacent organs, primarily kidney, bowel or pancreas was required in 83% of cases.[21] Despite such aggressive approaches, the rate of gross total resection is about 40–50% of all patients who present for resection.[1,21,28,29] Of 500 patients who presented at MSKCC, only 64% of primary tumors and 52% of recurrent sarcomas were grossly resected.[30] Among patients presenting with primary tumors that were resected with grossly negative margins, median overall survival was 103 months vs 18 months for those with primary tumors in which gross residual disease remained. There was no significant difference in survival between patients who were unresectable and those who underwent incomplete resection.

Aside from the extent of resection, the prognostic value of other clinicopathological factors are controversial. Most studies analyzing prognostic factors have evaluated the impact primarily of grade, histology, tumor stage and presentation status.[1,21,25–27,30–33] In the majority of these studies, high-grade lesions appear to confer a worse survival by as much as 30–70% compared with low-grade lesions;[21,26,27,31–33] however, studies from several major centers have not demonstrated any impact of grade on survival.[22–24,28] Among the studies that evaluate whether grade impacts on patterns of failure,[28,30,34–36] four suggest that high-grade tumors are associated with an increased incidence of distant metastases with three of them showing no impact on local failure while one found that high-grade designation increased the risk for local failure. On the other hand, two studies suggest that high-grade tumor status has no influence on the rates of distant metastases.[1,22] Lewis reported recently an analysis of prognostic factors based on data of 231 MSKCC patients with primary disease undergoing GTR.[30] High-grade status was associated with worse disease-specific mortality [relative risk (RR)=3.2, $p$=0.001], higher risk for distant metastases (RR=5, $p$=0.01) and increased local failure (RR=2, $p$=0.01). However, follow-up is short with a median time of 2.3 years. With median follow-up of 6.3 years, the data from Princess Margaret Hospital (PMH) demonstrated no survival impact of grade.[23] The experience from Mayo suggested that the apparent influence of grade on survival in an earlier report was lost with longer follow-up.[22]

Similar conflicting data exist for histological subtype and presentation, but not tumor stage. Five studies suggest that histological subtype has no impact on survival but four of these report that it affects the rate of distant metastases.[21,26,30,31,34] Two of these suggested that leiomyosarcoma is associated with a decrease rate of distant metastases,[30,34] but one reports the opposite effect of leiomyosarcoma.[21] Lewis reported that liposarcoma had a higher rate of local recurrence (RR=2.6, $p$=0.01) but did not impact on disease-specific survival most likely due to its negative association with metastasis (RR=0.2, $p$=0.01). In contrast, two studies report that histological subtype can impact on survival, both associating liposarcoma with improved survival.[23,37] Recurrent presentation status had no statistically significant impact on survival in one study[22] but did in another.[30] Tumor size is reported consistently not to influence survival[21,31,33,34] or risk for local failure.[31,33] However, T3 lesions (based on the Russell staging system) which invade adjacent organs have been reported to impact negatively on survival.[31,33] Of 198 patients who were followed for at least 5 years, Heslin reported that patients alive at 5 years tended to present with primary low-grade, liposarcomas in which GTR was achieved.[34]

### Patterns of failure after gross total resection

After GTR, patterns of failure studies indicate that local relapse represents the primary mode of failure. However, local failure after complete resection occurs in 41–77% (Table 1). In the largest such study from MSKCC, local failure alone as a first site of recurrence occurred in 81% of all failures and was a component of 90% of failures.[21] Distant metastases to lung or liver accounted for 19% of all first failures. Local failure alone comprised a higher proportion of first failures for those presenting with recurrent disease compared to those presenting with primary disease (92% vs. 67%, respectively). In a literature review of 310 patients achieving gross total resection, Fein showed that 47% recurred locally while 21% developed distant metastases.[28] Of 135 recurrent cases, 81% had a local component of failure and 37% had a distant component. Common life-threatening complications resulting from local recurrence include sepsis, gastrointestinal bleeding, obstruction, perforation, fistula, biliary obstruction and obstructive nephropathy.[32] Due to the high local failure rates despite maximal surgery, the role of adjuvant treatment becomes crucial.

### Adjuvant external beam radiation

Based on the highly successful experience in extremity sarcoma, it is clear that radiation delivered either by external beam radiotherapy (EBRT) or brachytherapy (BT) can serve as effective adjuvant treatment for sarcoma.[7,8,10,16,20] To control grossly resected extremity sarcomas, EBRT doses of 60–70 Gy or BT doses of 45 Gy are needed.[7,8,16,20] Combined BT (15–20 Gy) plus EBRT (45–50 Gy) have also been used.[10] Achieving such doses to treat retroperitoneal sarcomas is problematic due to the radiation tolerance of the surrounding organs as well as the large areas that need to be addressed. Tepper's study suggested that a post-operative EBRT dose greater than >60 Gy produced better local control 83% (5/6) vs 33% (2/6) for <50 Gy.[38] Catton showed that the addition of radiation increased the time to in-field local failure from 30 to 103 months (p<0.05), especially if a dose of >35 Gy was delivered.[23] Fein showed that of 21 retroperitoneal sarcoma patients, local failure was 25% (2/8) vs 38% (5/13) if the dose delivered was <55.2 Gy vs >55.2 Gy.[28] Cody demonstrated an increase in 5-year survival rates from 30 to 53% in 15 patients who received adjuvant radiation after GTR compared to 22 who had GTR alone; however, this was not statistically significant and a detailed comparison of the treatment groups was not performed.[32] Heslin showed that among 5-year survivors, radiation therapy was the only significant factor on univariate analysis that decreased local recurrence.[34]

Delivering doses greater than 50–55 Gy with standard EBRT technique is toxic. In the NCI trial, 54–55 Gy produced a 50% chronic enteritis and 25% fistula rate.[29] Glenn reported a severe enteritis rate of 22% (8/37) in patients receiving a dose of 54 Gy after GTR, of whom seven required surgery and one died from bowel perforation.[33] Tissue expanders to displace bowel as well as various radiation therapy treatment techniques (decubitis position, oblique fields) may improve tolerance to modest doses (50–55 Gy) but do not in general permit therapeutic doses. Preoperative radiation has been advocated to improve resectability and minimize bowel toxicity; however, its efficacy remains to be demonstrated and achieving doses for optimal control is not feasible.[22,39]

### Clinical experience with IORT in the treatment of retroperitoneal sarcoma

The addition of IORT to maximal resection and EBRT represents an attractive approach to delivering more effective adjuvant treatment. To date, there has been only a limited experience with this approach (Table 2). A randomized trial at the NCI showed significantly better local control with IOERT and post-operative EBRT 40% (6/15) vs post-operative EBRT alone 80% (16/20) $p<0.05$ at a median follow-up of 8 years.[29] All patients underwent gross total resection and were randomized to either EBRT of 35–40 Gy and 20 Gy IORT with misonidazole or to 50–55 Gy of EBRT alone. The IOERT arm experienced significantly more peripheral neuropathy (60% vs 5%, $p<0.05$), while the EBRT only arm had a significantly higher rate of gastrointestinal complications, including a higher rate of disabling chronic enteritis [50% (10/20) vs 7%(2/15), respectively] and fistula [25% (5/20) vs 0%(0/15), respectively]. The higher incidence of peripheral neuropathy in the IORT arm was attributed to multiple factors including the dose of 20 Gy, the use of a concomitant radiosensitizer which itself is neurotoxic, and the use of large pelvic electron fields near the lumbosacral plexus.

The MGH experience with IOERT was also favorable (Table 2).[39] Of 20 patients receiving preoperative EBRT to 40–50 Gy, 14 had a complete resection. Ten of the 14 completely resected patients received IORT to a median dose of 15 Gy (10–15 Gy) for microscopic residual tumor, while the other four received no further because of extensive tumor beds that could not be encompassed in an IORT field. At a median follow-up of 3 years, only 10% (1/10) who underwent GTR and IORT failed locally. Seven of 10 are NED, while three developed metastasis. None of the 10 patients undergoing IORT and GTR developed neuropathy. One of two patients who received IORT and EBRT for gross residual disease developed a sensory neuropathy after receiving 17.5 Gy of IORT. Bowel toxicity was minimal with only a 6% small bowel obstruction rate. At the same institution in a cohort of patients treated with resection and postoperative EBRT without IORT, the 5-year local control and survival rates were both 54%. Thus, the addition of IORT appeared to improve local control and possibly survival.

Other important limited experiences with IOERT have been reported (Table 2). The Radiation Therapy Oncology Group (RTOG) conducted a phase II study

**Table 2**. *Efficacy of IORT in the treatment of retroperitoneal sarcoma*

| | TX Date | No. of patients | Med F/U (years) | %GTR | %HG | RT Dose | 5 year LR (actuarial, %) | 5 year OS (actuarial, %) |
|---|---|---|---|---|---|---|---|---|
| Mayo Clinic[22] | 1981–97 | 88 | 3.5 | 81 | 62 | 48.6 Gy EBRT+ 15 Gy IORT | 41 | 47 |
| NCI[29] | NS | 20 | | 100 | NS | 54–55 Gy EBRT | 80 | 25 |
| | | | 8 | | | vs | [8-year] | [8-year] |
| | | 15 | | | | | $p$=0.05 | |
| | 15 | | | | | 35–40 Gy EBRT+ | 40 | 20 |
| | | | | | | 20 Gy IORT | [8-year] | [8-year] |
| MSKCC[35] | 1992–96 | 32 | 2.8 | 84 | 62 | 45 Gy EBRT+ 15 Gy IORT | 38 [4-year] | 56 [4-year DFS] |
| Institut Bergonie[41] | 1991–94 | 19 | 1.4 | 74 | 74 (Grade 2,3) | 50 Gy EBRT+ 17 Gy IORT | 24 [2-year] | 60 [2-year DFS] |
| MGH[39] | 1981–89 | 17 | 3 | 82 | 76 | 40–50 Gy EBRT+ 15–20 Gy IORT [59%] | 19 [4 year] | 64 [4-year DFS] |
| RTOG[40] | NS | 12 | 1.5 | NS | | 40–50 Gy EBRT+ 12.5–20 Gy IORT | 17 [2 year] | |

GTR=gross total resection, HG=high grade, LR=local recurrence, OS=overall survival, DFS=disease free survival, NCI=National Cancer Institute, MGH=Massachusetts General Hospital, NS=not stated, MSKCC=Memorial Sloan-Kettering Cancer Center, RTOG=Radiation Therapy Oncology Group.

of intraoperative radiation for retroperitoneal sarcomas.[40] A preliminary analysis of 12 patients treated with EBRT (45–50.4 Gy) plus IOERT (12.5–20 Gy) demonstrated a local failure of 17% at a median follow-up of 18 months. At the Institut Bergonie, 19 retroperitoneal sarcomas were treated with IOERT after maximal resection. GTR was achieved in 79% (15/19).[41] The median IOERT dose was 17 Gy (15–20 Gy) with 13/19 receiving EBRT at a median dose of 50 Gy (30–60 Gy). At a median follow-up of 17 months, 2-year actuarial local failure was 24% with 2-year overall survival of 60%. Severe late complications occurred in 6/19 patients and were likely multifactorial in origin with one 'moderate' peripheral neuropathy and one iliac vessel rupture.

Petersen at the Mayo Clinic reported the largest experience with IOERT in the treatment of 87 retroperitoneal sarcoma who had received maximal resection and preoperative EBRT.[22] Eighty-three per cent (72/87) were able to undergo GTR with 64% having microscopic residual and 20% had no residual tumors. All primary tumors (43/87) and 77% of recurrent tumors received a preoperative EBRT dose of 45–48.6 Gy. All patients received intraoperative electron radiation to a median dose of 15 Gy. At a median follow-up of 3.5 years, 23% (20/87) developed a local recurrence with a 3- and 5-year actuarial local recurrence of 23 and 41%, respectively. The amount of residual disease significantly affected local control with 5-year local failure of 0% for no residual, 43% for microscopic residual and 63% for gross residual tumors ($p$=0.04). Interestingly, local relapse occurred in only 7% (3/43) of primary tumors treated with IORT, EBRT and GTR. Five-year overall survival after gross total resection was 49%. Grade 3 peripheral neuropathy occurred in 10% (9/87) while grade 3 gastrointestinal toxicities occurred in 14% (12/87) consisting mainly of fistula and proctitis. No grade 4 toxicities were reported.

The preliminary Memorial Sloan-Kettering Cancer Center experience using HDR-IORT has been reported and is also favorable.[35,36] In a prospective protocol, 32 patients were treated with gross total resection and HDR-IORT (12–15 Gy) followed by post-operative EBRT (45–50.4 Gy). HDR-IORT was delivered using a cable-mounted iridium-192 source into a superflab afterloading applicator. Twelve patients presented with primary and 20 with locally recurrent disease. Two-thirds of the patient (20/32) had high-grade disease and the median tumor size was 20×12.5×11 cm. At a median follow-up of 33 months (range 1–77 months), the 4-year actuarial local failure for all patients was 38%. Subset analysis demonstrated that local failure for primary tumors was 26% vs 46% in recurrent disease ($p$=0.4). Tumor grade did not impact on the rate of local failure (40% vs 33% for high- and low-grade tumors, respectively, $p$=0.66). A statistically significant higher 4-year actuarial rate of distant metastases was detected in the high-grade vs low-grade tumors (30% vs 0%, $p$=0.05, respectively). Four-year actuarial disease-free and overall survival were 55 and 45%, respectively. Neither presentation status nor tumor grade impacted on disease-free or overall survival. In this challenging group of patients treated with an aggressive combined modality regimen, 34% developed complications, the majority of which were multifactorial in etiology and resolved with conservative management. Bowel obstruction was the most common complication (18%) and fistula formation occurred in 9%. Also noteworthy was a 6% (2/32)

incidence of femoral nerve palsy which were mild and healed without major intervention.

The low rate of peripheral neuropathy is reassuring. Based on animal studies and the NCI randomized trial, the main toxicity associated with IORT is peripheral neuropathy.[42] A dose of 20 Gy appears to be toxic as significant rates of peripheral neuropathy developed in the NCI trial (60%), while doses of 15 Gy or less appeared to be better tolerated (36%). One of the potential problems with IOERT is the dependence on unwieldy, rigid electron cones to treat narrow, anatomically complex surfaces. In addition, if the target is large, abutting electron fields must be used. These factors introduce dosimetric inhomogeneities that may underdose the target or overdose adjacent normal tissue. In the NCI trial, the most common situation associated with the development of clinically debilitating peripheral neuropathy was the use of angled, abutting electron fields to treat large pelvic tumors. Although the significantly lower rate of peripheral neuropathy noted in the MSKCC series may be due to a different patient population or differences in shielding techniques, it possibly is related to the type of applicator used. In contrast to the electron cone, the Harrison–Anderson–Mick (HAM) applicator used in the MSKCC study is flexible enough to conform to most tumor beds and can be abutted without junctional dosimetric inhomogeneity. A low rate of peripheral neuropathy was also reported in rectal cancer patients treated with HDR-IORT using the HAM applicator in a separate MSKCC study.[43–45]

### Conclusion

In summary, the preponderance of the data support the hypothesis that IORT can improve local control. When compared to the local recurrence rates of 41–82% after gross total resection without IORT (Table 1), the five studies evaluating this modality, including a randomized trial, indicate that IORT does appear to decrease local failure to rates of 19–41% (Table 2). However, with the exception of the 8-year follow-up of the randomized NCI trial, follow-up of most of the other studies is modest. The importance of long-term observation was demonstrated by Heslin, who showed that local failure can commonly occur even in 5-year survivors at up to 5% per year.[34] Thus, close surveillance is required to confirm the benefit of IORT on local control. Nevertheless, IORT appears to improve local control in other sites including colorectal, pancreatic and gastric cancers.[46]

Theoretically, since local recurrence represents the primary mode of failure and underlies the cause of death in the majority of retroperitoneal sarcoma patients, it would seem that a survival benefit should be derived from the incorporation of IORT. Yet an obvious improvement in survival is not apparent. It may be that too few patients treated with IORT have been studied as data of only 183 patients have been reported or it is possible that follow-up is too short to make a firm conclusion. The improvement in local control without a survival benefit has been demonstrated for extremity sarcomas.[20] Moreover, given the morbidity and mortality associated with local failure, local control remains a worthy objective. IORT seems to be a promising new modality for the 50% of patients whose retroperitoneal sarcomas may be gross totally resected; however, its role in subtotally resected patients remains to be defined. New treatment approaches integrating IORT, possibly concurrently with new chemotherapeutic or other biological agents need to be investigated.

### References

1 Storm FK, Mahvi DM. Diagnosis and management of retroperitoneal soft-tissue sarcoma. *Ann Surg* 1991; 214:2–10.

2 Ellis F. Tumor bed implants at the time of surgery, removable interstitial implants with iridium-192. In: *2nd International Symposium on Radiation Therapy*. New York: Memorial Sloan-Kettering Cancer Center, 1975.

3 Gemer LS, *et al.* Local recurrence of soft tissue sarcoma following brachytherapy. *Int J Radiat Oncol Biol Phys* 1991; 20:587–92.

4 Hilaris B, *et al.* Perioperative brachytherapy and surgery in soft tissue sarcomas. In: *Brachytherapy Oncology*. New York: Memorial Sloan-Kettering Cancer Center, 1982.

5 Hilaris B, *et al.* Limb-sparing therapy for locally advanced soft tissue sarcomas. *Endocur Hypertherm Oncol* 1985; 1:17–24.

6 Karakousis CP, *et al.* Feasibility of limb salvage and survival in soft tissue sarcomas. *Cancer* 1986; 57:484–91.

7 Lindberg R, *et al.* Conservative surgery and radiation therapy for soft tissue sarcomas. In: *Management of primary bone and soft tissue tumors*. Chicago, IL: Year Book Medical, 1977, 289–298.

8 Rosenberg SA, *et al.* Prospective randomized evaluation of the role of limb-sparing surgery, radiation therapy, and adjuvant chemoimmunotherapy in the treatment of adult soft-tissue sarcomas. *Surgery* 1978; 84:62–9.

9 Roy J, *et al.* Adjuvant endocurietherapy in the management of liposarcomas of the extremeties. *Endocur Hypertherm Oncol* 1990; 2:29–35.

10 Schray MF, *et al.* Soft tissue sarcoma. Integration of brachytherapy, resection, and external irradiation. *Cancer* 1990; 66:451–6.

11 Shiu MH, *et al.* Control of locally advanced extremity soft tissue sarcomas by function- saving resection and brachytherapy. *Cancer* 1984; 53:1385–92.

12 Shiu MH, *et al.* Brachytherapy and function-saving resection of soft tissue sarcoma arising in the limb. *Int J Radiat Oncol Biol Phys* 1991; 21:1485–92.

13 Suit HD, Russell WO, Martin RG. Management of patients with sarcoma of soft tissue in an extremity. *Cancer* 1973; 31:1247–55.

14 Suit HD, Russell WO, Martin RG. Sarcoma of soft tissue: clinical and histopathologic parameters and response to treatment. *Cancer* 1975; 35:1478–83.

15 Suit HD, *et al.* Preoperative, intraoperative, and postoperative radiation in the treatment of primary soft tissue sarcoma. *Cancer* 1985; 55:2659–67.

16 Suit HD, *et al.* Treatment of the patient with stage M0 soft tissue sarcoma. *J Clin Oncol* 1988; 6:854–62.

17 Willet C, Suit H. Limited surgery and external beam irradiation in soft tissue sarcoma. *Adv Oncol* 1989; 5:26–29.

18 Zelefsky MJ, *et al*. Limb salvage in soft tissue sarcomas involving neurovascular structures using combined surgical resection and brachytherapy. *Int J Radiat Oncol Biol Phys* 1990; 19:913–8.
19 Zelefsky MJ, *et al*. Combined surgical resection and iridium 192 implantation for locally advanced and recurrent desmoid tumors. *Cancer* 1991; 67:380–4.
20 Harrison LB, *et al*. Long-term results of a prospective randomized trial of adjuvant brachytherapy in the management of completely resected soft tissue sarcomas of the extremity and superficial trunk. *Int J Radiat Oncol Biol Phys* 1993; 27:259–65.
21 Jaques DP, *et al*. Management of primary and recurrent soft-tissue sarcoma of the retroperitoneum. *Ann Surg* 1990; 212:51–9.
22 Petersen I, *et al*. Use of intraoperative electron beam radiation therapy in the management of soft tissue sarcoma. In press, 1999.
23 Catton CN, *et al*. Outcome and prognosis in retroperitoneal soft tissue sarcoma. *Int J Radiat Oncol Biol Phys* 1994; 29:1005–10.
24 van Doorn RC, *et al*. Resectable retroperitoneal soft tissue sarcomas. The effect of extent of resection and postoperative radiation therapy on local tumor control. *Cancer* 1994; 73:637–42.
25 Singer S, *et al*. Prognostic factors predictive of survival for truncal and retroperitoneal soft-tissue sarcoma. *Ann Surg* 1995; 221:185–95.
26 McGrath PC, *et al*. Improved survival following complete excision of retroperitoneal sarcomas. *Ann Surg* 1984; 200:200–4.
27 Kilkenny JW, 3rd, Bland KI, Copeland EM, 3rd. Retroperitoneal sarcoma: the University of Florida experience. *J Am Coll Surg* 1996; 182:329–39.
28 Fein DA, *et al*. Management of retroperitoneal sarcomas: does dose escalation impact on locoregional control? *Int J Radiat Oncol Biol Phys* 1995; 31:129–34.
29 Sindelar WF, *et al*. Intraoperative radiotherapy in retroperitoneal sarcomas. Final results of a prospective, randomized, clinical trial. *Arch Surg* 1993; 128:402–10.
30 Lewis JJ, *et al*. Retroperitoneal soft-tissue sarcoma: analysis of 500 patients treated and followed at a single institution. *Ann Surg* 1998; 228:355–65.
31 Dalton RR, *et al*. Management of retroperitoneal sarcomas. *Surgery* 1989; 106:725–32; discussion 732–3.
32 Cody HSD., *et al*. The continuing challenge of retroperitoneal sarcomas. *Cancer* 1981; 47:2147–52.
33 Glenn J, *et al*. Results of multimodality therapy of resectable soft-tissue sarcomas of the retroperitoneum. *Surgery* 1985; 97:316–25.
34 Heslin MJ, *et al*. Prognostic factors associated with long-term survival for retroperitoneal sarcoma: implications for management. *J Clin Oncol* 1997; 15:2832–9.
35 Hu K, Harrison LB. High dose-rate intraoperative radiation therapy (HDR-IORT) for retroperitoneal sarcomas. In: *21st Annual Meeting of the American Brachytherapy Society*. San Diego, CA, 1999.
36 Alektiar KM, *et al*. High dose-rate intraoperative brachytherapy (HDR-IORT) for retroperitoneal sarcomas. In press.
37 Zhang G, *et al*. Sarcomas of the retroperitoneum and genitourinary tract. *J Urol* 1989; 141:1107–10.
38 Tepper JE, *et al*. Radiation therapy of retroperitoneal soft tissue sarcomas. *Int J Radiat Oncol Biol Phys* 1984; 10:825–30.
39 Willett CG, *et al*. Intraoperative electron beam radiation therapy for retroperitoneal soft tissue sarcoma. *Cancer* 1991; 68:278–83.
40 Kiel KD, *et al*. Preliminary results of protocol RTOG 85-07: phase II study of intraoperative radiation for retroperitoneal sarcomas. In: *3rd International Symposium of Intraoperative Radiation Therapy*. Oxford: Pergamon Press, 1991.
41 Bussieres E, *et al*. Retroperitoneal soft tissue sarcomas: a pilot study of intraoperative radiation therapy. *J Surg Oncol* 1996; 62:49–56.
42 Shaw EG, *et al*. Peripheral nerve and ureteral tolerance to intraoperative radiation therapy: clinical and dose-response analysis. *Radiother Oncol* 1990; 18:247–55.
43 Harrison LB, Enker WE, Anderson LL. High-dose-rate intraoperative radiation therapy for colorectal cancer. *Oncology (Huntingt)* 1995; 9:737–41; discussion 742–8 *passim*.
44 Harrison LB, Enker WE, Anderson LL. High-dose-rate intraoperative radiation therapy for colorectal cancer. *Oncology (Huntingt)* 1995; 9:679–83.
45 Harrison LB, *et al*. High dose rate intraoperative radiation therapy (HDR-IORT) as part of the management strategy for locally advanced primary and recurrent rectal cancer. *Int J Radiat Oncol Biol Phys* 1998; 42:325–30.
46 Gunderson LL, *et al*. Intraoperative irradiation: current and future status. Semin Oncol 1997; 24:715–31.

## References

[PDF_00665] Bussières E., Stöckle E. P., Richaud P. M., Avril A. R., Kind M. M., Kantor G., Coindre J. M., Nguyen Bui B. (1996). Retroperitoneal soft tissue sarcomas: a pilot study of intraoperative radiation therapy.. J Surg Oncol.

[PDF_00610] Catton C. N., O'Sullivan B., Kotwall C., Cummings B., Hao Y., Fornasier V. (1994). Outcome and prognosis in retroperitoneal soft tissue sarcoma.. Int J Radiat Oncol Biol Phys.

[PDF_00637] Cody H. S., Turnbull A. D., Fortner J. G., Hajdu S. I. (1981). The continuing challenge of retroperitoneal sarcomas.. Cancer.

[PDF_00635] Dalton R. R., Donohue J. H., Mucha P., van Heerden J. A., Reiman H. M., Chen S. P. (1989). Management of retroperitoneal sarcomas.. Surgery.

[PDF_00626] Fein D. A., Corn B. W., Lanciano R. M., Herbert S. H., Hoffman J. P., Coia L. R. (1995). Management of retroperitoneal sarcomas: does dose escalation impact on locoregional control?. Int J Radiat Oncol Biol Phys.

[PDF_00545] Gemer L. S., Trowbridge D. R., Neff J., Lin F., Reddy E., Evans R. G., Hassanein R. (1991). Local recurrence of soft tissue sarcoma following brachytherapy.. Int J Radiat Oncol Biol Phys.

[PDF_00639] Glenn J., Sindelar W. F., Kinsella T., Glatstein E., Tepper J., Costa J., Baker A., Sugarbaker P., Brennan M. F., Seipp C. (1985). Results of multimodality therapy of resectable soft-tissue sarcomas of the retroperitoneum.. Surgery.

[PDF_00683] Gunderson L. L., Willett C. G., Harrison L. B., Petersen I. A., Haddock M. G. (1997). Intraoperative irradiation: current and future status.. Semin Oncol.

[PDF_00675] Harrison L. B., Enker W. E., Anderson L. L. (1995). High-dose-rate intraoperative radiation therapy for colorectal cancer.. Oncology (Williston Park).

[PDF_00671] Harrison L. B., Enker W. E., Anderson L. L. (1995). High-dose-rate intraoperative radiation therapy for colorectal cancer.. Oncology (Williston Park).

[PDF_00599] Harrison L. B., Franzese F., Gaynor J. J., Brennan M. F. (1993). Long-term results of a prospective randomized trial of adjuvant brachytherapy in the management of completely resected soft tissue sarcomas of the extremity and superficial trunk.. Int J Radiat Oncol Biol Phys.

[PDF_00678] Harrison L. B., Minsky B. D., Enker W. E., Mychalczak B., Guillem J., Paty P. B., Anderson L., White C., Cohen A. M. (1998). High dose rate intraoperative radiation therapy (HDR-IORT) as part of the management strategy for locally advanced primary and recurrent rectal cancer.. Int J Radiat Oncol Biol Phys.

[PDF_00642] Heslin M. J., Lewis J. J., Nadler E., Newman E., Woodruff J. M., Casper E. S., Leung D., Brennan M. F. (1997). Prognostic factors associated with long-term survival for retroperitoneal sarcoma: implications for management.. J Clin Oncol.

[PDF_00604] Jaques D. P., Coit D. G., Hajdu S. I., Brennan M. F. (1990). Management of primary and recurrent soft-tissue sarcoma of the retroperitoneum.. Ann Surg.

[PDF_00554] Karakousis C. P., Emrich L. J., Rao U., Krishnamsetty R. M. (1986). Feasibility of limb salvage and survival in soft tissue sarcomas.. Cancer.

[PDF_00623] Kilkenny J. W., Bland K. I., Copeland E. M. (1996). Retroperitoneal sarcoma: the University of Florida experience.. J Am Coll Surg.

[PDF_00632] Lewis J. J., Leung D., Woodruff J. M., Brennan M. F. (1998). Retroperitoneal soft-tissue sarcoma: analysis of 500 patients treated and followed at a single institution.. Ann Surg.

[PDF_00620] McGrath P. C., Neifeld J. P., Lawrence W., DeMay R. M., Kay S., Horsley J. S., Parker G. A. (1984). Improved survival following complete excision of retroperitoneal sarcomas.. Ann Surg.

[PDF_00560] Rosenberg S. A., Kent H., Costa J., Webber B. L., Young R., Chabner B., Baker A. R., Brennan M. F., Chretien P. B., Cohen M. H. (1978). Prospective randomized evaluation of the role of limb-sparing surgery, radiation therapy, and adjuvant chemoimmunotherapy in the treatment of adult soft-tissue sarcomas.. Surgery.

[PDF_00568] Schray M. F., Gunderson L. L., Sim F. H., Pritchard D. J., Shives T. C., Yeakel P. D. (1990). Soft tissue sarcoma. Integration of brachytherapy, resection, and external irradiation.. Cancer.

[PDF_00668] Shaw E. G., Gunderson L. L., Martin J. K., Beart R. W., Nagorney D. M., Podratz K. C. (1990). Peripheral nerve and ureteral tolerance to intraoperative radiation therapy: clinical and dose-response analysis.. Radiother Oncol.

[PDF_00574] Shiu M. H., Hilaris B. S., Harrison L. B., Brennan M. F. (1991). Brachytherapy and function-saving resection of soft tissue sarcoma arising in the limb.. Int J Radiat Oncol Biol Phys.

[PDF_00571] Shiu M. H., Turnbull A. D., Nori D., Hajdu S., Hilaris B. (1984). Control of locally advanced extremity soft tissue sarcomas by function-saving resection and brachytherapy.. Cancer.

[PDF_00629] Sindelar W. F., Kinsella T. J., Chen P. W., DeLaney T. F., Tepper J. E., Rosenberg S. A., Glatstein E. (1993). Intraoperative radiotherapy in retroperitoneal sarcomas. Final results of a prospective, randomized, clinical trial.. Arch Surg.

[PDF_00617] Singer S., Corson J. M., Demetri G. D., Healey E. A., Marcus K., Eberlein T. J. (1995). Prognostic factors predictive of survival for truncal and retroperitoneal soft-tissue sarcoma.. Ann Surg.

[PDF_00538] Storm F. K., Mahvi D. M. (1991). Diagnosis and management of retroperitoneal soft-tissue sarcoma.. Ann Surg.

[PDF_00586] Suit H. D., Mankin H. J., Wood W. C., Gebhardt M. C., Harmon D. C., Rosenberg A., Tepper J. E., Rosenthal D. (1988). Treatment of the patient with stage M0 soft tissue sarcoma.. J Clin Oncol.

[PDF_00583] Suit H. D., Mankin H. J., Wood W. C., Proppe K. H. (1985). Preoperative, intraoperative, and postoperative radiation in the treatment of primary soft tissue sarcoma.. Cancer.

[PDF_00577] Suit H. D., Russell W. O., Martin R. G. (1973). Management of patients with sarcoma of soft tissue in an extremity.. Cancer.

[PDF_00580] Suit H. D., Russell W. O., Martin R. G. (1975). Sarcoma of soft tissue: clinical and histopathologic parameters and response to treatment.. Cancer.

[PDF_00654] Tepper J. E., Suit H. D., Wood W. C., Proppe K. H., Harmon D., McNulty P. (1984). Radiation therapy of retroperitoneal soft tissue sarcomas.. Int J Radiat Oncol Biol Phys.

[PDF_00657] Willett C. G., Suit H. D., Tepper J. E., Mankin H. J., Convery K., Rosenberg A. L., Wood W. C. (1991). Intraoperative electron beam radiation therapy for retroperitoneal soft tissue sarcoma.. Cancer.

[PDF_00596] Zelefsky M. J., Harrison L. B., Shiu M. H., Armstrong J. G., Hajdu S. I., Brennan M. F. (1991). Combined surgical resection and iridium 192 implantation for locally advanced and recurrent desmoid tumors.. Cancer.

[PDF_00592] Zelefsky M. J., Nori D., Shiu M. H., Brennan M. F. (1990). Limb salvage in soft tissue sarcomas involving neurovascular structures using combined surgical resection and brachytherapy.. Int J Radiat Oncol Biol Phys.

[PDF_00649] Zhang G., Chen K. K., Manivel C., Fraley E. E. (1989). Sarcomas of the retroperitoneum and genitourinary tract.. J Urol.

[PDF_00613] van Doorn R. C., Gallee M. P., Hart A. A., Gortzak E., Rutgers E. J., van Coevorden F., Keus R. B., Zoetmulder F. A. (1994). Resectable retroperitoneal soft tissue sarcomas. The effect of extent of resection and postoperative radiation therapy on local tumor control.. Cancer.

